# Observations on American Medical Practice

**Published:** 1808-05

**Authors:** 


### *To the Editors of the Medical and Physical Journal.*

GENTLEMEN,

I Have been a constant reader of the Lond. Med. and Phys. Journal from the commencement of its publication, and an occasional contributor to its contents; proofs that I have hitherto approved of the mode of conducting it.

It is with no small degree of pain, that I now hear frequent complaints among my medical acquaintance, of a want of selection of cases of cautious practice, and that admittance is too frequently afforded to dangerous trans-atlantic communications. Not that I would interdict, Gentlemen, the reception of *foreign* intelligence from your Journal, or wish to confine you solely to indigenous literature; but no thinking man, or conscientious practitioner of physic, can readily bring himself to believe, that the *Herculean* practice of our American brethren, which has of late occupied the pages of your Journal, can be imitated with impunity in *our island.* The recital of cases lately transmitted to you can answer no good purpose; on the contrary, it is fraught with the greatest danger.* Your Journal is very widely circulated amongst

---

* The Editors are of opinion, that it answers a good purpose to show what can be done in other climates, as well as the United Kingdom; and that their readers possess sufficient discrimination to guard them against the contagion of rash practice.

British practitioners, and perused more particularly by the *junior part* of the profession, who anxiously wait the revolving period of its publication, in the hopes of adding something to their stock of knowledge, that might lead them to success in their future practice. What then must be their dissappointment, Gentlemen, on perusing its contents, where the most prominent feature is the *Brunonian theory* reduced to practice with a vengeance? Numerous instances might be adduced from your latter Journals, some of which, (if I recollect right) have already been animadverted upon by some more rational practitioner at home. But too much cannot be said here, in condemnation of American practice.

If they chuse to salivate in phthissis pulmonalis, and at the same time draw blood to the amount of *thirty ounces* daily, for several days together, and deplete in every possible manner; let them enjoy the felicity of seeing their patients escape with life, after putting in force such unwarrantable practice; but why allow them to infuse these murderous principles among us?

I cannot deny that such is their practice, but I can deny that it would be alike successful in our island. The American mode of living may be different from ours, their stamina more robust; but from such depleting doctors may my countrymen be ever defended.

The patients account of himself, from whom I have quoted the above *notable* method of practice,* is, that in the spring of 1803, he was attacked with phthisis pulmonalis, and as advised by an eminent physician, immediately began to rub in strong *mercurial ointment*, and to take calomel in as *large doses* as his constitution would bear.

Now, considering this was an *American* constitution, these doses no doubt would astonish a *British* practitioner; but the relator cruelly forbears to inform us whether each dose contained ten, twenty, or thirty grains of calomel, but I think we may venture to place it not less than the first; he was "*previously* reduced by *night* sweats and a plentiful expectoration;" to add to his misery, he was attacked with peripneumony! In forty-eight hours the friction and calomel produced ptyalism, for he very justly remarks, that under

* Vide Med. and Phys. Journal for March, 1808, in a Letter to Dr. Rush, page 219.

under the circumstances above mentioned, there was *no time to be lost!* so that "*thirty ounces* of blood were taken away daily, for several days together," to give the "mercury liberty to operate," a very judicious reason for venesection. So soon as his mouth became affected, "the cough ceased, expectoration abated, and *every* pulmonaty symptom began to disappear." In September of the same year, however, he adds, "*Now* I cough none, of *any consequence*, and do not expectorate more than two or three times a day." Yet this is a case of phthisis pulmonalis *cured* by mercury: and *venesection* should likewise have been added. Yet he is "at a loss to know, whether he should *continue the ptyalism*, or have *recourse to tonics*," although in the very next line he adds, "my *debility* is still *very great*, not being yet able to walk alone." Surprising!

I have only, Gentlemen, to apologize to *you* for troubling you with these observations, assuring you they are meant as a friendly hint; the above case *can* require no comment: to state the facts as they are related is sufficient for the discriminating eye of the generality of your readers; I shall therefore only add, that by turning over the pages of your former Journals, *other cases* can be pointed out where the *mode of* treatment was still more preposterous, and unjustifiable upon any solid scientific than that of the preceding, and where the patients were not quite so fortunate, but who were doomed to fall victims to obstinate perseverance in that grand catholicon of transatlantic art, copious depletion.

I am, &c.

A PRACTITIONER IN WILTSHIRE.

*April* 5, 1808.

*Dr.* KENTISH'S

